# The Value of Prognostic Nutritional Index (PNI) in Predicting Survival and Guiding Radiotherapy of Patients With T1-2N1 Breast Cancer

**DOI:** 10.3389/fonc.2019.01562

**Published:** 2020-01-30

**Authors:** Xin Hua, Zhi-Qing Long, Xin Huang, Jia-Peng Deng, Zhen-Yu He, Ling Guo, Wen-Wen Zhang, Huan-Xin Lin

**Affiliations:** ^1^Sun Yat-sen University Cancer Center, State Key Laboratory of Oncology in South China, Collaborative Innovation Center for Cancer Medicine, Guangzhou, China; ^2^Department of Radiotherapy, Sun Yat-sen University Cancer Center, Guangzhou, China; ^3^Department of Nasopharyngeal Carcinoma, Sun Yat-sen University Cancer Center, Guangzhou, China

**Keywords:** prognostic nutritional index, breast cancer, prognostic factor, radiotherapy, survival

## Abstract

**Background:** To investigate the significance of the prognostic nutrition index (PNI) as a predictor of survival and guide for treating T1-2N1 breast cancer.

**Methods:** Patients with T1-2N1 breast cancer (*N* = 380) who underwent a mastectomy at our center were studied. PNI was calculated as 10 × serum albumin (g/dl) + 0.005 × total lymphocyte count (per mm^3^). The cutoff for the PNI was calculated using the time-dependent receiver operating characteristic (ROC) curve analysis by overall survival (OS) prediction. The associations between the PNI and the clinicopathologic characteristics were analyzed using Pearson's χ^2^ test. Survival curves were calculated using the Kaplan–Meier method. Univariate and multivariate analyses were performed using the Cox proportional hazards model.

**Results:** Subgroup analyses of patients with low PNI value (≤52.0) and high PNI value (>52.0) showed that a high PNI was significantly associated with HER2 status, the neutrophil–lymphocyte ratio (NLR), the monocyte–lymphocyte ratio (MLR), and KI 67 status. The OS of patients with a high PNI was significantly better than that of patients with a low PNI. We then conducted subgroup analyses based on PNI and radiotherapy. Among patients who received radiotherapy, the OS of those with a high PNI was significantly better than that of patients with a low PNI. Among patients with a high PNI, the OS of those who received radiotherapy was better than that of the patients who did not receive radiotherapy. However, among the patients with a low PNI, the OS of those who received radiation was worse than that of patients who did not receive radiotherapy. The Kaplan–Meier survival analysis and the multivariate analysis of patients with T1-2N1 breast cancer who received radiotherapy showed PNI independently predicted OS.

**Conclusions:** The preoperative PNI may be a reliable predictor of OS of patients with operable T1-2N1 breast cancer, with the capacity to provide a personalized prognosis and facilitate the development of clinical treatment strategies. However, radiotherapy did not achieve satisfactory outcomes in patients with PNI ≤52.0; thus, further studies on treatment optimization are needed.

## Background

Breast cancer is one of the most common malignancies among women worldwide and those in China (1, 2). Although a comprehensive treatment model for breast cancer has been established and the concept of precision medicine has become increasingly popular with more standardized and accurate treatments and rapidly updated guidelines, unresolved disputes persist after years of debate. As an important component of systematic treatment, radiotherapy remains controversial for its indications, and there is no consensus on whether all patients with T1-2N1 breast cancer need radiotherapy (3, 4). According to the National Comprehensive Cancer Network (NCCN) Guidelines, postoperative patients with T3-4N1 breast cancer require radiotherapy of the chest wall and the supraclavicular/subclavian lymph node drainage area, to reduce local recurrence rates and improve overall survival (OS) (5). However, the NCCN Guidelines do not specify whether radiotherapy is necessary for patients with T1-2N1 breast cancer. Therefore, patient stratification and individualized precision therapies are needed.

The question of whether prophylactic postoperative radiotherapy should be used to treat patients with T1-2N1 breast cancer has a long history as a source of controversy. The Early Breast Cancer Trialists' Collaborative Group suggested that postoperative radiotherapy reduces local recurrence and distant metastasis of T1-2N1 breast cancer (3). Some studies have found that postoperative adjuvant radiotherapy does not reduce local recurrence or improve survival among patients with T1-2N1 breast cancer (6). Given some of the inevitable side effects of radiotherapy, several studies have recommended that postoperative adjuvant radiotherapy for patients with T1-2N1 breast cancer should be avoided because of insufficient evidence (7). Therefore, further research is urgently needed to identify novel biomarkers associated with prognosis to provide accurate predictions of T1-2N1 breast-cancer prognoses.

In recent years, greater attention has been paid to the influence of nutrition and immune status on patients' prognosis of cancer. The prognostic nutrition index (PNI) is calculated based on the serum albumin concentration and peripheral blood lymphocyte count, and is an indicator of the nutritional and immune status of cancer patients (8). Many studies have found that PNI is an independent prognostic indicator of various malignant tumors (9–11). Mohri et al. had found that PNI was an effective marker to predict the long-term prognosis in breast cancer (12). Also, similar results have been found in triple-negative breast cancer (13). However, as far as we know, research on the prognostic value of this marker for patients with T1-2N1 breast cancer undergoing controversial therapeutic regimens has not been conducted. Therefore, this retrospective study examined the predictive value of the preoperative PNI for survival outcomes of patients with T1-2N1 breast cancer.

## Materials and Methods

### Patients

Between December 2010 and November 2012, a retrospective study was conducted with 380 patients who underwent surgery for invasive breast cancer at Sun Yat-sen University Cancer Center (SYSUCC). Histopathological and clinical examination data were obtained for all patients. The inclusion criteria were as follows: (1) female breast carcinoma; (2) histopathological examination confirmed the diagnosis of invasive breast cancer and no distant metastasis; (3) received radical/modified radical mastectomy. The exclusion criteria were as follows: (1) breast conserving surgery or male breast carcinoma; (2) treatment with neoadjuvant chemotherapy or radiotherapy; (3) presence of distant metastasis; (4) the number of dissected axillary lymph node ≤10; or (5) incomplete laboratory data. All patients were pathological staged using the 7th edition of the American Joint Committee on Cancer-Tumor, Node and Metastases (AJCC-TNM) staging system.

### Data Collection and Definitions

Primary preoperative laboratory data from within 3 days of the time of surgery and clinicopathologic data were retrieved from the patients' medical records. ER, PR, p53, Ki-67, and HER2 expression were determined using immunohistochemistry (IHC) and expression status was defined as described previously (14). The PNI was calculated (12) as 10 × serum albumin (g/dl) + 0.005 × total lymphocyte count (per mm^3^). The neutrophil–lymphocyte ratio (NLR), platelet–lymphocyte ratio (PLR), and monocyte–lymphocyte ratio (MLR) were calculated (15) as follows: NLR = N/L; PLR = P/L; and MLR = M/L, where P, N, M, and L represent the platelet (10^9^/L), neutrophil (10^9^/L), monocyte (10^9^/L), and lymphocyte (10^9^/L) counts, respectively.

### Treatment

Patients included in this study underwent mastectomy with axillary lymph node dissection or sentinel lymph node biopsy. Most of them received systematic therapy as adjuvant chemotherapy, endocrine treatment, or Trastuzumab for human epidermal growth factor receptor-2 (Her-2)-positive tumor. RT of the chest wall and regional nodes was applied with a dose prescription of 50 Gy in 25 fractions. All patients received a three-dimensional conformal radiotherapy technique with opposed tangential beams to the chest wall or whole breast.

### Follow-Up

The patients were followed up with an outpatient examination or a telephone interview. OS was defined as the time from the date of surgery to the date of death or the final follow-up.

### Ethics Statement

This study was approved by the Research Ethics Committee of SYSUCC and all patients provided written informed consent.

### Statistical Analyses

Statistical analyses were performed using SPSS 23.0 (SPSS Inc., Chicago, IL), R project version 3.4.1, and GraphPad Prism 6.0 software (GraphPad, La Jolla, CA). The cutoff for the PNI was calculated using the time-dependent receiver operating characteristic (ROC) curve analysis by the total OS and the highest Youden index for predicting OS. Time-dependent ROC was depicted using the Kaplan–Meier method via the survival ROC package in R. The associations between the PNI and the clinicopathologic characteristics were analyzed using Pearson's χ^2^ test or Fisher's Exact Test. Survival curves were calculated using the Kaplan–Meier method and compared using the log-rank test. Univariate and multivariate analyses were performed using the Cox proportional hazards model. Two-tailed *P*-values < 0.05 were considered statistically significant.

## Results

### Patient Characteristics

The optimal cutoff points that were calculated using the time-dependent ROC by OS prediction for the PNI, NLR, PLR, and MLR are shown in Table 1. The baseline characteristics of the study's 380 patients are shown in Table 2. The median PNI was 55.90 (range = 38.50–85.00). The median age of the patients was 47 years (range = 26–78 years), and the median follow-up period was 63.1 months (range = 3.2–95.9 months). During the final follow-up, 28 (7.4%) patients died and 352 (92.6%) survived.

**Table 1:** Receiver operating characteristics analyses of parameters of inflammation in patients with T1-2N1 breast cancer.

**Variables**	**Cutoff value**	**AUC (95% CI)**	**Specificity**	**Sensitivity**
PNI	51.625	0.598 (0.491–0.705)	0.665	0.536
NLR	1.9307	0.535 (0.422–0.647)	0.554	0.571
PLR	645.22	0.534 (0.417–0.652)	0.437	0.714
MLR	0.134	0.523 (0.403–0.644)	0.676	0.536

**Table 2:** Clinicopathologic characteristics of patients with T1-2N1 breast cancer (*N* = 380).

**Characteristic**	**No. of patients (%)**
**Age (years)**	
≤60	328 (86.3)
>60	52 (13.7)
**Histological type**	
Invasive ductal carcinoma	355 (93.4)
Others	25 (6.6)
**T stage**	
T1	129 (33.9)
T2	251 (66.1)
**Estrogen receptor**	
Positive	270 (71.1)
Negative	106 (27.9)
Unknown	4 (1.1)
**Progesterone receptor**	
Positive	248 (65.3)
Negative	128 (33.7)
Unknown	4 (1.1)
**HER2**	
Positive	110 (28.9)
Negative	239 (62.9)
Unknown	31 (8.2)
**p53**	
Positive	231 (60.8)
Negative	132 (34.7)
Unknown	17 (4.5)
**Ki-67**	
Positive	247 (65.0)
Negative	122 (32.1)
Unknown	11 (2.9)
**CEA**	
Positive	27 (7.1)
Negative	339 (89.2)
Unknown	14 (3.7)
**CA15-3**	
Positive	48 (12.6)
Negative	318 (83.7)
Unknown	14 (3.7)
**Adjuvant chemotherapy**	
Yes	331 (87.1)
No	49 (12.9)
**Adjuvant radiotherapy**	
Yes	108 (28.4)
No	272 (71.6)
**Endocrine therapy**	
Yes	186 (48.9)
No	194 (51.1)
**NLR**	
≤1.93	207 (54.5)
>1.93	173 (45.5)
**PLR**	
≤645	162 (42.6)
>645	218 (57.4)
**MLR**	
≤0.13	129 (33.9)
>0.13	251 (66.1)
**PNI**	
≤52	133 (35.0)
>52	247 (65.0)

### Relationships Between the PNI and Clinicopathologic Characteristics

The cutoff value for the PNI was 52; 133 patients had low PNI, whereas 247 patients had a high PNI. As shown in Table 3, the treatment patterns between the two groups were comparable (adjuvant chemotherapy, *P* = 0.712; adjuvant radiotherapy, *P* = 0.215; endocrine therapy, *P* = 0.846), and a high PNI was significantly associated with HER2 status (*P* = 0.044), NLR (*P* < 0.001), MLR (*P* = 0.006), and KI 67 status (*P* = 0.013).

**Table 3:** Associations of the PNI with the clinicopathologic characteristics of patients with T1-2N1 breast cancer.

**Feature**	**Total (*N* = 380)**	**PNI value**		***P***
		**Low**	**High**	
**Age (years)**				
≤60	328 (86.3%)	115 (35.1%)	213 (64.9%)	0.950
>60	52 (13.7%)	18 (34.6%)	34 (65.4%)	
**Histological type**				
Invasive ductal carcinoma	355 (93.4%)	128 (36.1%)	227 (63.9%)	0.104
Others	25 (6.6%)	5 (20.0%)	20 (80.0%)	
**T classification**				
1	129 (33.9%)	46 (35.7%)	83 (64.3%)	0.847
2	251 (66.1%)	87 (34.7%)	164 (65.3%)	
**ER**				
Negative	106 (27.9%)	35 (33.0%)	71 (67.0%)	0.595
Positive	270 (71.1%)	97 (35.9%)	173 (64.1%)	
**PR**				
Negative	128 (33.7%)	42 (32.8%)	86 (67.2%)	0.503
Positive	248 (65.3%)	90 (36.3%)	158 (63.7%)	
**HER2**				
Negative	239 (62.9%)	94 (39.3%)	145 (60.7%)	**0.044**
Positive	110 (28.9%)	31 (28.2%)	79 (71.8%)	
**Ki-67**				
Positive	247 (65.0%)	77 (31.2%)	170 (68.8%)	**0.013**
Negative	122 (32.1%)	54 (44.3%)	68 (55.7%)	
**p53**				
Positive	231 (60.8%)	83 (35.9%)	148 (64.1%)	0.836
Negative	132 (34.7%)	46 (34.8%)	86 (65.2%)	
**CEA**				
Positive	27 (7.1%)	11 (40.7%)	16 (59.3%)	0.493
Negative	339 (89.2%)	116 (34.2%)	223 (65.8%)	
**CA15-3**				
Positive	48 (12.6%)	17 (35.4%)	31 (64.6%)	0.911
Negative	318 (83.7%)	110 (34.6%)	208 (65.4%)	
**Adjuvant chemotherapy**				
Yes	331 (87.1%)	117 (35.3%)	214 (64.7%)	0.712
No	49 (12.9%)	16 (32.7%)	33 (67.3%)	
**Adjuvant radiotherapy**				
Yes	108 (28.4%)	43 (39.8%)	65 (60.2%)	0.215
No	272 (71.6%)	90 (33.1%)	182 (66.9%)	
**Endocrine therapy**				
Yes	186 (48.9%)	66 (35.5%)	120 (64.5%)	0.846
No	194 (51.1%)	67 (34.5%)	127 (65.5%)	
**PLR**				
≤645	162 (42.6%)	54 (33.3%)	108 (66.7%)	0.557
>645	218 (57.4%)	79 (36.2%)	139 (63.8%)	
**NLR**				
≤1.93	207 (54.5%)	56 (27.1%)	151 (72.9%)	**<0.001**
>1.93	173 (45.5%)	77 (44.5%)	96 (55.5%)	
**MLR**				
≤0.13	129 (33.9%)	33 (25.6%)	96 (74.4%)	**0.006**
>0.13	251 (66.1%)	100 (39.8%)	151 (60.2%)	

*Bold values means the P-value is significant*.

### Prognostic Analyses of the PNI in Patients With T1-2N1 Breast Cancer

**Figure 1:**
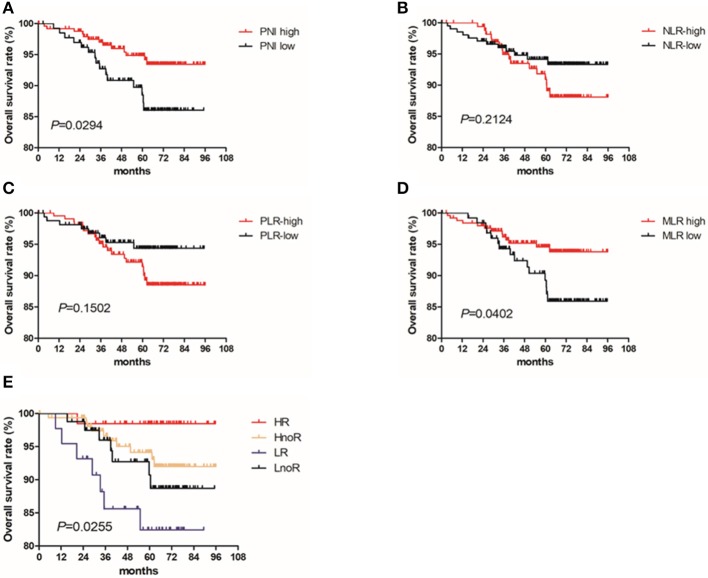
Kaplan Meier survival curves for OS by PNI and treatment with radiotherapy for patients with T1-2N1 breast cancer. Kaplan–Meier curves for overall survival: **(A)** stratified by PNI; **(B)** stratified by NLR; **(C)** stratified by PLR; **(D)** stratified by MLR; **(E)** stratified by PNI and radiotherapy.

The 5-year OS rates for patients with high and low PNIs were 94.9% and 87.3% (*P* = 0.029), respectively. Only the PNI and MLR were statistically significant in the OS analysis (Figures 1A–D), and the PNI was a superior predictor compared to the commonly recognized indices of systemic inflammation: the NLR, PLR, and MLR. The OS in patients with a high PNI was significantly longer than that of patients with a low PNI (*P* = 0.029; Figure 1A).

### Univariate and Multivariate Cox Regression Analysis of OS among Patients With T1-2N1 Breast Cancer

Univariate Cox regression analysis revealed that estrogen receptor (ER) status, progesterone receptor (PR) status, MLR, and the PNI all had significant associations with survival in patients with stage N1 breast cancer. Variables significant in univariate analysis and those most likely to be prognostic were involved in multivariate survival analysis and revealed that the PNI independently predicted the OS of patients with stage N1 breast cancer (*P* = 0.027; Table 4).

**Table 4:** Univariate and multivariate Cox regression analyses of the associations between the clinicopathologic characteristics and the overall survival of patients with T1-2N1 breast cancer.

**Characteristic**	**Univariate analysis**		**Multivariate Cox regression analysis**	
	**Hazards ratio (95% CI)**	***P***	**Hazards ratio (95% CI)**	***P***
Age (years)	1.058 (0.367–3.050)	0.917		
Histological type	0.577 (0.078–4.251)	0.590	0.407 (0.047–3.560)	0.417
T stage	1.835 (0.749–4.495)	0.184	1.316 (0.521–3.323)	0.561
ER	0.341 (0.160–0.727)	**0.005**	0.624 (0.173–2.243)	0.470
PR	0.363 (0.170–0.776)	**0.009**	0.597 (0.168–2.116)	0.424
HER2	1.361 (0.912–2.031)	0.131		
Ki-67	2.780 (0.958–8.067)	0.060	2.850 (0.938–8.663)	0.065
p53	1.340 (0.583–3.083)	0.491		
CEA	2.608 (0.898–7.574)	0.078	3.059 (0.950–9.855)	0.061
CA15-3	1.595 (0.604–4.214)	0.346		
Adjuvant radiotherapy	0.763 (0.324–1.796)	0.610		
Adjuvant chemotherapy	1.022 (0.308–3.389)	0.972		
Endocrine therapy	0.549 (0.256–1.174)	0.122		
NLR high vs. low	1.603 (0.758–3.388)	0.217	1.884 (0.788–4.504)	0.154
PLR high vs. low	1.810 (0.797–4.109)	0.156	1.322 (0.494–3.540)	0.578
MLR high vs. low	0.468 (0.223–0.984)	**0.045**	0.359 (0.140–0.920)	**0.033**
PNI high vs. low	0.448 (0.213–0.941)	**0.034**	0.396 (0.174–0.900)	**0.027**

*Bold values means the P-value is significant*.

### Prognostic Analyses of the PNI Subgroups

We divided the patients into four groups based on their PNIs and whether they received radiotherapy: (1) the HR (PNI high + radiotherapy) group, (2) the HnoR (PNI high + no radiotherapy) group, (3) the LnoR (PNI low + no radiotherapy) group, and (4) the LR (PNI low + radiotherapy) group. We further analyzed the effect of PNI and radiotherapy on the OS of the patients using Cox proportional hazards model and the Kaplan–Meier method. The OS of the HR group was better than that of the HnoR group [*P* = 0.036; Hazards ratio (95% CI) = 0.366 (0.081–1.652)]; the HnoR group's OS was better than that of the LnoR group [*P* = 0.033; Hazards ratio (95% CI) = 0.175 (0.035–0.868)]; and the LnoR group's OS was better than that of the LR group [*P* = 0.048; Hazards ratio (95% CI) = 0.807 (0.163–3.998)] (Figure 1E).

We compared survival differences by PNI (high and low) in patients with T1-2N1 breast cancer who received radiotherapy. Patients who received radiotherapy were divided into two groups based on their PNIs; the OS of patients with a high PNI was significantly better than that of patients with a low PNI (*P* = 0.008). The patients with a high PNI were divided into two groups based on whether they received radiotherapy. The OS of patients who received radiotherapy was better than that of the patients who did not receive it (*P* = 0.080).

### Univariate and Multivariate Cox Regression Analyses of OS Among Patients With T1-2N1 Who Received Radiotherapy

The univariate Cox regression analysis revealed that the cancer antigen 15-3 (CA15-3) biomarker test and the PNI were significantly associated with survival of the patients who received radiotherapy. A multivariate survival analysis revealed that the PNI independently predicted OS of the patients who received radiotherapy (*P* = 0.028; Table 5).

**Table 5:** Univariate and multivariate Cox regression analyses of the associations between the clinicopathologic characteristics and the overall survival of patients with T1-2N1 breast cancer treated with radiotherapy.

**Characteristic**	**Univariate analysis**		**Multivariate Cox regression analysis**	
	**Hazards ratio (95% CI)**	***P***	**Hazard ratio (95% CI)**	***P***
Age (years)	0.046 (0.000–2.4 × 10^5^)	0.697		
Histological type	1.960 (0.236–16.302)	0.533	6.291 (0.389–101.804)	0.195
T stage	1.955 (0.379–10.077)	0.423	2.150 (0.295–15.652)	0.450
ER	0.389 (0.078–1.927)	0.247		
PR	0.258 (0.047–1.410)	0.118		
HER2	4.438 (0.742–26.564)	0.103		
Ki-67	0.776 (0.142–4.239)	0.770		
p53	1.033 (0.189–5.641)	0.970		
CEA	4.500 (0.525–38.572)	0.170		
CA15-3	6.496 (1.311–32.189)	**0.022**	11.696 (1.774–77.136)	**0.011**
Adjuvant chemotherapy	21.279 (0.000–7.6 × 10^7^)	0.730		
Endocrine therapy	0.416 (0.093–1.860)	0.251		
NLR high vs. low	2.391 (0.464–12.325)	0.297	1.311 (0.189–9.107)	0.784
PLR high vs. low	0.668 (0.150–2.987)	0.598	0.254 (0.029–2.219)	0.215
MLR high vs. low	1.079 (0.209–5.564)	0.927	0.254 (0.029–2.219)	0.415
PNI high vs. low	0.099 (0.012–0.821)	**0.032**	0.047 (0.003–0.715)	**0.028**

*Bold values means the P-value is significant*.

## Discussion

This study was conducted to investigate the significance of the PNI as a predictor of clinical outcomes and guide for treating T1-2N1 breast cancer. Our results found that patients with a high PNI had longer OS than patients with a low PNI. Furthermore, among patients who received radiotherapy, the OS of those with a high PNI was significantly longer than that of patients with a low PNI. Among patients with a high PNI, the OS of those who received radiotherapy was longer than that of the patients who did not receive radiotherapy. However, among the patients with a low PNI, the OS of those who received radiation was shorter than that of patients who did not receive radiotherapy.

The PNI, a new systemic immune-nutrition index, represents the immune and nutritional status of the host and is a significant biomarker for various tumors (16, 17). Onodera initially developed the PNI to evaluate postoperative complications in patients undergoing gastrointestinal surgery (18). Subsequently, additional studies found that the PNI was closely related to the long-term prognoses of tumors and was an independent prognostic factor in the survival of patients with various cancers (9, 13, 16, 17). We conducted a retrospective study to explore the significance of the PNI in prognosis predictions of patients with T1-2N1 breast cancer. The present study showed that the PNI was an independent marker of survival among these patients and among patients with T1-2N1 breast cancer treated with radiotherapy. We also found that the PNI was a superior predictor compared to the commonly used indices of systemic inflammation, i.e., the NLR, PLR, and MLR.

There is no doubt that radiotherapy reduces the probability of postoperative local recurrence and improves the survival of patients with advanced tumor stages (19), although the use of radiotherapy to treat patients with early tumor stages of breast cancer is controversial. The NCCN Guidelines strongly recommend postoperative radiotherapy for patients with T1-2N1 breast cancer; whether postoperative radiotherapy should be prophylactic remains controversial. Three randomized clinical trials demonstrated that radiotherapy reduced local recurrence and improved survival in patients with one to three axillary lymph-node metastases (20, 21). However, these trials are 15–20 years old. Two of them used CMF chemotherapy regimens (anthracene ring, taxicyclines, and targeted drugs), which have not been used to treat breast cancer, and one of them used tamoxifen for only 1 year, whereas the use of tamoxifen is now recommended for at least 5 years. The number of lymphatic dissections was fewer than 7 in many of the included patients, while the current view is that at least 8 axillary lymph nodes should be dissected (21). The local recurrence rate in these trials was as high as 30%, suggesting that the intensity of treatment at the time was inadequate. Studies have also shown that postoperative adjuvant radiotherapy does not reduce local recurrence or improve the survival of patients with T1-2N1 breast cancer (6). Postoperative radiotherapy has been reported to reduce the probability of recurrence by 14% in patients with a tumor size of 2–5 cm accompanied by metastases to two to three axillary lymph nodes. However, it had no significant benefit in patients with a tumor size <2 cm accompanied by metastases to fewer than three axillary lymph nodes or a tumor size of 2–5 cm accompanied by metastasis to one lymph node (22). As radiotherapy has inevitable side effects that may affect patients' survival, studies recommend avoiding the use of postoperative adjuvant radiotherapy for patients with T1-2N1 breast cancer on grounds of insufficient evidence (7). In other words, it is possible that radiotherapy is not necessary to treat all patients with T1-2N1 breast cancer while treatment without radiotherapy might also be inadequate. In this study, 108 patients treated with radiotherapy and 247 patients with high PNI were included in the stratified analysis. Among patients treated with radiotherapy, those with high PNI had better OS than those with a low PNI. In contrast, patients with a low PNI who received radiotherapy had worse OS than those who did not receive radiotherapy. These results suggest that patients with a high PNI are more likely to benefit from radiotherapy and the PNI is a reliable host-related factor that is useful for further differentiating the heterogeneity of radiotherapy benefits among patients with T1-2N1 breast cancer.

The PNI was calculated based on serum albumin levels and the total number of lymphocytes in peripheral blood. Many studies have shown that the PNI represents cancer patients' nutritional and immune status and affects their survival (9, 10, 16). Serum albumin has been found to be an independent predictor of prognosis in several studies of patients with malignant tumors, including breast cancer (23–25). Previous studies have also found that circulating albumin has specific anti-tumor effects on breast cancer (26). Moreover, malnutrition affects the prognosis of patients by weakening immune functioning, and lymphocyte level is an indicator of cell-mediated immunity, which plays an important role in cancer prevention of the host (27). Malnutrition has been identified as a risk factor for poor prognoses of various malignant tumors because of increased side effects, decreased chemotherapy intensity, interruptions in treatment, reduced radiotherapy sensitivity and/or chemotherapy sensitivity, and impaired immune function (28–30). Although none of the patients in this study showed delayed or interrupted radiotherapy or decreased dose intensity of chemotherapy, we believe that the nutritional status of the patients with a low PNI may have continued to deteriorate during radiotherapy and chemotherapy, thereby reducing radio-sensitivity and/or chemical sensitivity and ultimately leading to a reduced survival rate. In other words, patients with a high PNI have better immune function and are sensitive to radiotherapy. The side effects of radiotherapy are relatively mild, so they can enjoy the therapeutic benefits of radiotherapy. In contrast, patients with a low PNI are not sensitive to radiotherapy because of impaired immune function, which is aggravated by the various side effects of radiotherapy; thus, radiotherapy is not worth its disadvantages. These findings might help clinicians gain a better understanding of the relationships between immunization, nutrition, and tumors, and guide the development of appropriate therapeutic regimens for individualized precision therapies in patients with T1-2N1 breast cancer.

This study has several limitations. First, conclusions from a retrospective study with a relatively small sample size may be biased. Second, the PNI is a nonspecific tumor marker because other non-cancer diseases can be confused with a tumor; therefore, further prospective studies are required to validate our findings. In addition, PNI could be affected by various pathological conditions, such as infection and medications, and varies from time to time. Therefore, we are going to collect more data in the hope of conducting dynamic analysis and obtaining more results, and we are also seeking data from other centers to confirm our result.

## Conclusions

In summary, the present study demonstrates that the PNI is a useful prognostic factor in T1-2N1 breast cancer, and patients with a high PNI are more likely to benefit from radiotherapy. The PNI has the potential to be used as a risk factor for the selection of radiotherapy for patients with T1-2N1 breast cancer, which may facilitate the development of personalized treatment strategies to improve the treatment outcomes of patients with breast cancer.

## Data Availability Statement

The data and materials of this study have been included at RDD (http://www.researchdata.org.cn/) with the number of RDDA2020001367.

## Ethics Statement

The studies involving human participants were reviewed and approved by the Research Ethics Committee of Sun Yat-sen University Cancer Center. The patients/participants provided their written informed consent to participate in this study.

## Consent for Publication

All authors approve the version to be published and agree to be accountable for all aspects of the work in ensuring that questions related to the accuracy or integrity of any part of the work are appropriately investigated and resolved.

## Author Contributions

XHua and H-XL: conceptualization and formal analysis. XHua and XHuan: methodology. XHuan and Z-QL: software. Z-QL: validation. Z-QL, XHua, and J-PD: investigation. H-XL and W-WZ: resources. XHua, H-XL, and LG: data curation. XHua: writing (original draft preparation). All authors: writing (review and editing). XHua, Z-YH, and LG: visualization. W-WZ and H-XL: supervision. H-XL: project administration. LG, H-XL, W-WZ, and Z-YH: funding acquisition.

### Conflict of Interest

The authors declare that the research was conducted in the absence of any commercial or financial relationships that could be construed as a potential conflict of interest.

## Abbreviations

PNI: Prognostic nutrition index
ALB: albumin
ROC: receiver operating characteristic
OS: overall survival
NLR: neutrophil–lymphocyte ratio
MLR: the monocyte–lymphocyte ratio
HR: PNI high + radiotherapy
HnoR: PNI high + no radiotherapy
LnoR: PNI low +no radiotherapy
LR: PNI low + radiotherapy
NCCN: National Comprehensive Cancer Network.
